# Lying down frequency as a discomfort index in heat stressed Holstein bull calves

**DOI:** 10.1038/s41598-018-33451-6

**Published:** 2018-10-10

**Authors:** Levente Kovács, Fruzsina L. Kézér, Mikolt Bakony, Viktor Jurkovich, Ottó Szenci

**Affiliations:** 1 MTA–SZIE Large Animal Clinical Research Group, Dóra major H-2225, Üllő, Hungary; 2 Institute of Animal Husbandry, Faculty of Agricultural and Environmental Science, Szent István University, Páter Károly utca 1, Gödöllő, H-2100 Hungary; 3 Department of Animal Hygiene, Herd Health and Veterinary Ethology, University of Veterinary Medicine, István utca 2, Budapest, H-1078 Hungary

## Abstract

Changes in lying behaviour in response to extreme ambient temperatures have not been examined in dairy calves so far. In this study, lying time, and frequency of lying down were investigated in shaded (n = 8) and non-shaded (n = 8) Holstein bull calves during a 5-d period [temperature, average/max (°C); Day 1 (control, all calves shaded): 22.9/29.4, Day 2 (heat stress day): 28.3/38.8, Day 3: 26.2/33.5, Day 4: 23.7/28.7, and Day 5: 21.2/24.7]. The thermal environment around the calves was characterized by the temperature–humidity index (THI). A three-dimension accelerometer was used to record posture of the calves and lying time and lying down frequency were analysed with 4-h sampling intervals. On Day 1 no differences were found in THI between the shaded and non-shaded environments. On Days 2, 3 and 4 maximal and average THI were higher in the shaded than those recorded for the non-shaded environment. On Day5 no significant differences in THI were observed between calf environments. A similar diurnal pattern of lying time and lying down frequency was observed in both groups. Lying times were shorter during the afternoon (*P* = 0.003); however, no group differences were found in lying time (*P* = 0.551). During the daytime (between 8:00 and 20:00), the frequency of lying down was 50, 33, and 41% higher, respectively, than during the nighttime on Days 2, 3 and 4 (*P* < 0.001, *P* = 0.011, and *P* < 0.001). On the heat stress day, non-shaded calves changed posture 88.4 and 76.6% more often than shaded ones between 8:00 and 12:00 and 12:00 and 16:00, respectively (*P* < 0.001 for both intervals). Similar group differences were observed for Day 3 between 8:00 and 12:00 (71.2%) and Day 4 between 12:00 and 16:00 (76.6%), respectively (*P* = 0.003, and *P* = 0.001). On Day 5, there was no difference between groups (*P* = 0.732). As indicated by our results, heat stress causes changes in lying down frequency and lying time in dairy calves. Supplemental shading reduces discomfort as indicated by lying down frequency, but not by lying time.

## Introduction

Global warming and related weather patterns are associated with unprecedentedly high ambient temperatures, which can increase morbidity and mortality in livestock animals, including dairy cattle^1^. Hutch-reared dairy calves are vulnerable to warm episodes during summer because their natural heat-dissipating behaviour (e.g. finding shade) is hindered. As these are all parts of complex comfort behaviour, total time spent lying down, the number of lying bouts, and the frequency of lying down are often studied in response to environmental factors in adult cattle^2,3^. Although the effect of in utero heat stress exposure on calf activity patterns^4^ and on overall well-being^5,6^ have been recently evaluated during the neonatal period, there is a paucity of information regarding biobehavioral responses to acute heat stress experienced by calves during the pre-weaning period. Commercial automatic data loggers overcome the limitation of time-consuming video-based behavioural observations in calves;^7^ however, no research has evaluated lying behaviour in dairy calves during extreme heat stress events.

Due to the common ‘row-feeding’ method of hutch-reared calves, hutches are often positioned in a face-off-arrangement (‘bull row’ vs. ‘heifer row’). This way, hutches of bull calves are often exposed to direct sunlight as farmers would like to protect heifer calves from heat load, therefore, bull calves are often at risk of heat stress. The aim of the present study was to assess lying behaviour of shaded (S) and non-shaded (NS) bull calves to determine the effect of heat stress on calf comfort. We hypothesized changes in lying behaviour in response to heat stress, and that calves in shaded hutches would show less discomfort (increased lying times and lower lying down frequencies) than calves housed in hutches without supplemental shade.

## Results and Discussion

On Day 1 (control) no group differences were found in maximal (74.2 ± 0.1 vs. 74.5 ± 0.1; *P* = 0.930) and daily average values for THI (69.8 ± 0.1 vs. 69.5 ± 0.1; *P* = 0.945). The THI recordings indicated an extreme heat load on Day 2 (heat stress day) and Day 3. On Day 2, maximal THI was observed at 16:00 in the NS hutch environment that was higher than those recorded for the S one (86.4 ± 0.1 vs. 78.2 ± 0.1; *P* < 0.001). Reduced heat load in the S hutch environment compared to the NS one was reflected by daily averages of THI as well on the heat stress day (78.1 ± 0.1 vs. 71.3 ± 0.1; *P* = 0.011). Similar reductions were found in ambient temperature^8,9^ and THI^10^ in shaded commercial calf hutches during short-term warm episodes in summer. Like on Day 2, maximal (82.3 ± 0.1 vs. 74.5 ± 0.1; *P* = 0.002) and average THI (74.7 ± 0.1 vs. 68.3 ± 0.1; *P* = 0.011) were higher in the NS than the S environment for Day 3 (*P* = 0.003 and *P* = 0.014). Similar, but moderate differences were found on Day 4 for maximal (78.5 ± 0.1 vs. 73.7 ± 0.1; *P* = 0.015) and average THI (71.4 ± 0.1 vs. 66.5 ± 0.1; *P* = 0.026). On Day 5 maximal and average THI for the NS and S hutch environments were 71.0 ± 0.1 vs. 69.5 ± 0.1 and 64.2 ± 0.1 vs. 63.7 ± 0.1 respectively, without significant differences (*P* = 0.526 and *P* = 0.545).

**Figure 1:**
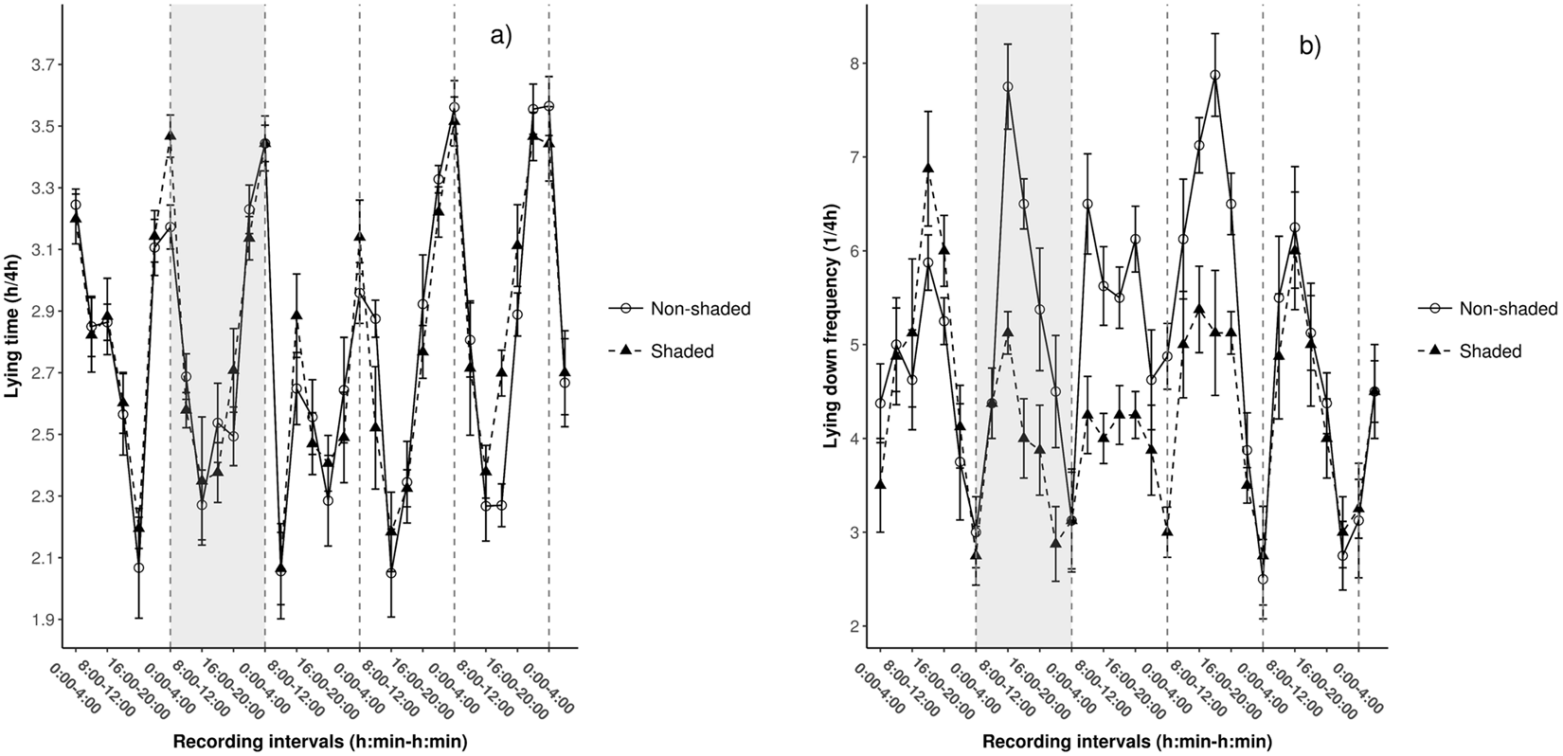
Changes in lying time (**a**) and lying down frequency (**b**) of shaded (▲, n = 8) and non-shaded (○, n = 8) dairy calves during the 5-d experiment. Data are presented as means ± SEM. During Day 1 (control), all calves were shaded. The grey area between the first and the second dashed vertical lines represents the “heat stress day”.

Diurnal patterns of lying time and lying down frequency were observed in both groups throughout the experimental period (Fig. 1a,b). Calves changed posture more frequently during the afternoon that resulted in shorter lying times. Lying time tended to decrease during daytime in both groups and increased during the afternoon and evening periods to reach its higher values between 0:00 and 4:00 at night (Fig. 1a). On Days 2 and 3 lying time decreased rapidly for the period between 4:00 and 8:00 (*P* < 0.001 for both days and groups). On Days 4 and 5 similar lengths of lying time were observed to Days 1 and 2. The frequency of lying down showed similar patterns between groups during Day 1 (control), with 32% higher values for daytime compared to nighttime (*P* = 0.003). During the daytime (between 8:00 and 20:00), the frequency of lying down was 50, 33, and 41% higher, respectively, than during the nighttime on Days 2, 3 and 4 (*P* < 0.001, *P* = 0.011, and *P* < 0.001, respectively). Besides discomfort caused by heat stress, diurnal changes in ambient temperature and anticipation around the time of afternoon feeding^11^ are possibly reflected in the observed activity patterns.

It was hypothesized that calves in a S environment would show less discomfort than calves exposed to a NS environment. Our hypothesis was supported by significant increases in the frequency of lying down in NS calves during the daytime periods on Days 2, 3, and 4. On Day 1 no effect of shading was observed on the frequency of lying down (*P* = 0.551). On the heat stress day, NS calves changed posture 88.4 and 76.6% more often than S ones between 8:00 and 12:00 and 12:00 and 16:00, respectively (*P* < 0.001 for both intervals). Similar group differences were observed also for Day 3 between 8:00 and 12:00 (71.2%) and Day 4 between 12:00 and 16:00 (76.6%), respectively (*P* = 0.003, and *P* = 0.001). Increased daytime lying down frequency in NS calves during the most heat straining periods illustrates short-term acclimatory responses^12^ initiated to compensate for increased heat stress. On Day 5, calves changed posture 44% more often during the daytime compared to nighttime (*P* < 0.001); however, we found no group difference in lying down frequency (*P* = 0.732). No study evaluated lying behaviour of calves exposed to heat stress; however, in accordance to the present findings, it was observed that in case of thermal discomfort adult dairy cattle change posture more often to maximize heat dissipation from the body surface^13,14^.

Opposite to the finding for lying down frequency, no differences were found between S and NS calves for lying time throughout the 5-d period. Though lying down frequency was higher in NS calves, the durations of standing bouts were relatively short that did not significantly reduce overall lying time. Due to the larger surface area/mass ratio in calves compared to adult cattle, heat dissipation of young animals might be not considerably larger in a standing posture. On the other hand, NS calves preferred hutches to pens in sunny hours and were usually in a lying posture due to limited space. Based on these observations lying time as behavioural measure of heat stress has limitations when used in calves housed in individual hutches.

Our study provided novel data on heat stress-induced changes in lying behaviour of dairy calves. In conclusion, lying time and lying down frequency show a diurnal pattern in hutch-reared dairy calves that was possibly affected by acute heat stress. Supplemental shading reduced the frequency of lying down on days with maximal THI above 78 but did not affect lying time in this study. As the first response of animals to stress is the behavioural one, which is the most efficient way to try to cope with the situation^15^, automatic recording of lying down frequency may be a promising approach for early detection of acute heat stress in dairy calves. Further research on behavioural patterns during acute heat stress events would aid in the development of housing for young calves in warm climate regions.

## Methods

### Animals and experimental design

All methods and the applied procedures on the animals were performed in accordance with the relevant guidelines and regulations of the Pest County Government Office, Department of Animal Health (Permit Number: PE/EA/1973-6/2016) that approved the study. The experiment was carried out on a large-scale dairy farm in Hungary (N47°18′191′′ E18°48′336′′), which has a herd of over 1000 lactating Holstein cows. The farm was visited for a 6-day period in the month of August [temperature; average/min/max (°C): 28.9/22.6/38.8, measured in shadow]. Preweaned Holstein bull calves (n = 16) at one month of age with a mean body weight of 74 (±3.2) kg were used for the study. Calves were housed in 1.65 × 1.20 m individual hutches with a 1.60 m^2^ exercise pen in front of each hutch. Hutches and pens had concrete floors bedded with straw. Experimental hutches were aligned in one row, oriented north to south.

The experiment covered 6 days and was designed and timed based on the expected temperature patterns of the regional weather forecast. Day 0 (habituation to the study environment) was followed by Day 1 (control day, temperature; average/max (°C): 20.7/26.8), Day 2 (“heat stress day”, temperature; average/max (°C): 27.8/38.8) and Days 3–5: (“post-stress period”, temperature; average/max (°C); Day 3: 26.2/33.5, Day 4: 23.7/28.7, Day 5: 21.2/24.7). Between 8:00 and 14:00 on Day 0, a shading structure was prepared over 16 adjacent hutches and pens where experimental calves were kept. A sun shade net with shade rate of 85% was used as shading material and was located 1.9 m above the ground. The following 10 h served as a habituation period for the calves to get used to the novel environment. At 24:00 on Day 1, the shading net was removed from 8 adjacent hutches that remained exposed to direct sunlight throughout the experiment (NS, n = 8), while the other 8 adjacent hutches remained shaded (S, n = 8) for the reminder of the study period. Both S and NS calves were fed the same diet, which did not change throughout the experiment. Calves received one feeding of 2 L of milk replacer at 6:00 and 18:00 and had ad libitum access to chopped alfalfa hay and the starter grain diet (Purina calf starter, Cargill, USA), which met the requirements for preweaned Holstein calves^16^. Water from a plastic bucket (7.6 liters), filled twice a day, was provided throughout the study. The diet did not change throughout the experiment.

### Recording of the thermal environment

Thermal environment around the animals was assessed by recording the ambient temperature and relative humidity at 10-min intervals. Data loggers were fitted inside the hutches (VOLTCRAFT DL-181THP, Conrad Electronic SE, Hirschau, Germany). Daily maxima and averages of the temperature-humidity index (THI) were used to characterize the microclimatic environment:^17^

$$THI=(0.35\times Tdb+0.65\times Twb)\times 1.8+32$$

where T_db_ = dry bulb temperature and T_wb_ = wet bulb temperature.

This THI was recommended by Bohmanova *et al*.^18^ for “low-relative-humidity” (continental) climatic regions.

### Assessment of lying behaviour

A three-dimension accelerometer (HOBO Pendant G data logger, Onset Computer Corporation, Bourne, MA) was used to record body posture. The logger was attached to the right hind leg of the calves^7^. Data was downloaded using Onset HOBOware Software (Onset Computer Corporation, Bourne, MA) and exported to Microsoft Excel. The data logger recorded the g force on the *x*, *y*, and *z*-axes on a scale of −1 to 1. The cut-off values used to categorize logger readings as a specific behaviour (lying vs. standing) were determined based on preliminary observation from video recordings. Information from the *x*-axis was used to evaluate lying down (g < 0.75) and standing (g ≥ 0.75) with a 30-s sampling interval, considering that lying down and standing bouts are short^19^. The time spent lying down (lying time) and the frequency of lying down were calculated for 4-h recording intervals and presented as means plus standard error of the mean (SEM).

### Statistical analyses

Statistical analyses were performed in the R–3.3.1 statistical environment and language^20^. To evaluate the effect of shading on the thermal environment, daily maximal and average values of THI were calculated for all experimental days using all recorded meteorological data with 10-min sampling frequency. Comparisons between S and NS environments were made with the *t*-test at the significance level of 0.05. Daily variations in lying time were compared using linear mixed models, with group (S vs. NS) and 4-h sampling periods as fixed factors. Due to the Poisson distribution of data and the relatively low sample size, lying down frequency was compared between groups by fitting Poisson regression to GLM models. The model included group and time of day (daytime: 8:00–20:00; nighttime: 20:00–8:00) as fixed factors. All models analyzed behavioural data included calf within treatment as a random factor. Comparison of S and NS groups were made with the Fisher-type *z*-transformation-based *z*-test in all models. A *P*-value < 0.05 was considered significant. All results are expressed as mean plus SEM values.

## Data Availability

All materials, data and associated protocols are available to readers any restrictions.
